# Kynurenic acid and cancer: facts and controversies

**DOI:** 10.1007/s00018-019-03332-w

**Published:** 2019-10-28

**Authors:** Katarzyna Walczak, Artur Wnorowski, Waldemar A. Turski, Tomasz Plech

**Affiliations:** 1 Department of Pharmacology, Medical University of Lublin, Chodźki 4a, 20-093 Lublin, Poland; 2 Department of Biopharmacy, Medical University of Lublin, Chodźki 4a, 20-093 Lublin, Poland; 3 Department of Experimental and Clinical Pharmacology, Medical University of Lublin, Jaczewskiego 8, 20-090 Lublin, Poland

**Keywords:** Cancer therapy, Proliferation, Cell cycle, GPR35, AhR

## Abstract

Kynurenic acid (KYNA) is an endogenous tryptophan metabolite exerting neuroprotective and anticonvulsant properties in the brain. However, its importance on the periphery is still not fully elucidated. KYNA is produced endogenously in various types of peripheral cells, tissues and by gastrointestinal microbiota. Furthermore, it was found in several products of daily human diet and its absorption in the digestive tract was evidenced. More recent studies were focused on the potential role of KYNA in carcinogenesis and cancer therapy; however, the results were ambiguous and the biological activity of KYNA in these processes has not been unequivocally established. This review aims to summarize the current views on the relationship between KYNA and cancer. The differences in KYNA concentration between physiological conditions and cancer, as well as KYNA production by both normal and cancer cells, will be discussed. The review also describes the effect of KYNA on cancer cell proliferation and the known potential molecular mechanisms of this activity.

## Introduction

**Fig. 1:**
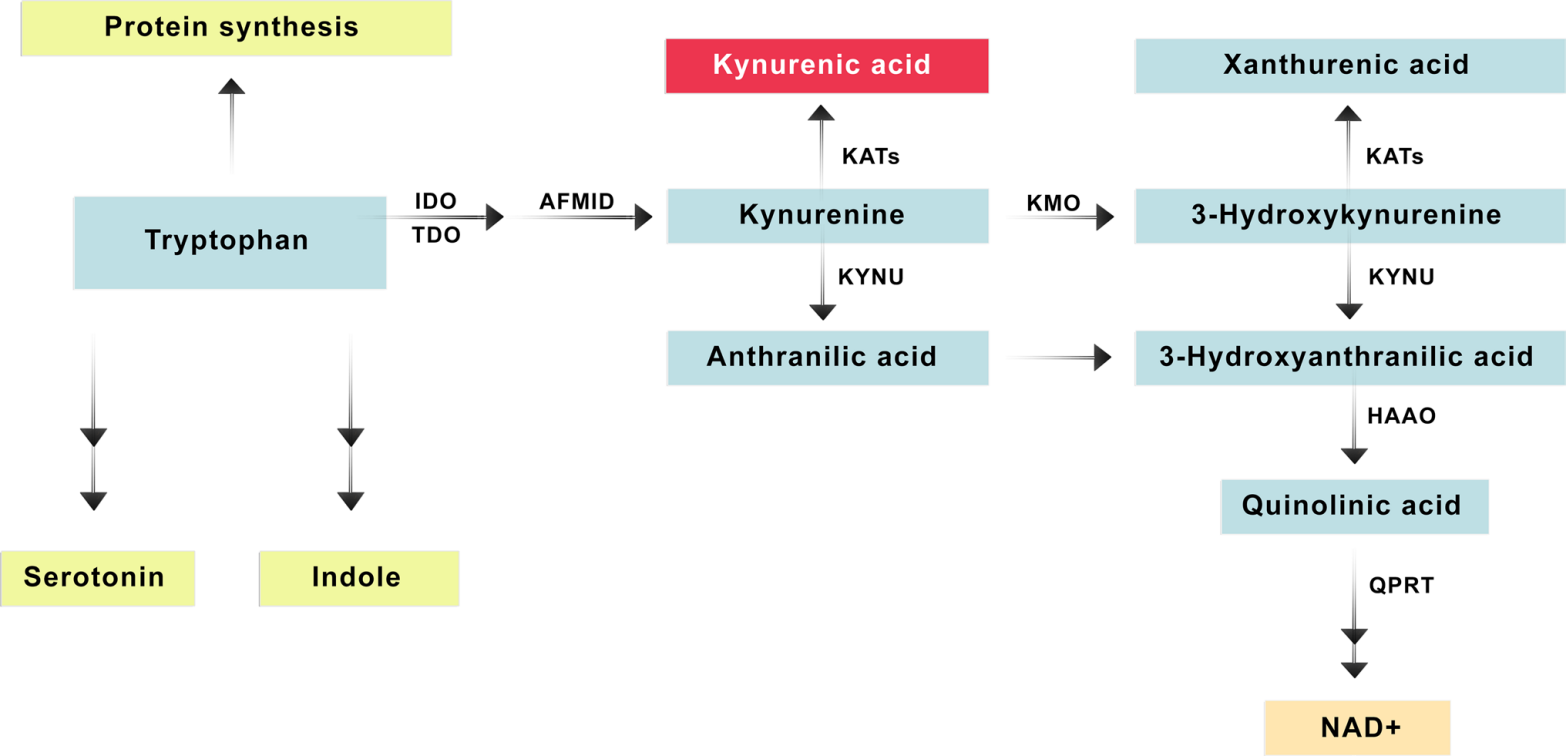
The kynurenine pathway of tryptophan degradation. The selected enzymes of the kynurenine pathway are shown in a simplified scheme. *AFMID* kynurenine formamidase (arylformamidase), *HAAO* 3-hydroxyanthranilate 3,4-dioxygenase, *IDO* indoleamine-2,3-dioxygenase, *KATs* kynurenine aminotransferases [includes four isoenzymes: KAT I (*KYAT1*), KAT II (*AADAT*), KAT III (*KYAT3*), KAT IV (*GOT2*)], *KMO* kynurenine-3-monooxygenase, *KYNU* kynureninase, *QPRT* quinolinate phosphoribosyl transferase, *TDO* tryptophan 2,3-dioxygenase, *NAD* nicotinamide adenine dinucleotide

Tryptophan is a precursor to many biologically active compounds in addition to its irreplaceable role in the process of protein biosynthesis. The kynurenine pathway is the principal route of catabolism of tryptophan, leading to the formation of the important redox cofactor nicotinamide adenine dinucleotide (NAD^+^) and several biologically active metabolites. However, tryptophan is also metabolized along the serotonin pathway leading to the biosynthesis of neuroactive substances: serotonin and melatonin. Importantly, it is also a substrate for gut bacterial production of indoles which are potentially involved in signalling between microbiota and innate immune system (Fig. 1) [1–3].

Previous studies have suggested that deregulation of the kynurenine pathway may lead to cancer progression through decreasing the effectiveness of the antitumour immune response (reviewed in [4, 5]). The contribution of enzymes involved in tryptophan degradation: indoleamine 2,3-dioxygenase (IDO) and tryptophan 2,3-dioxygenase (TDO) has been previously discussed (reviewed in [4, 5]). Expression of IDO1 is considered as a negative prognostic marker in several types of cancer including colon cancer, melanoma, haematological and gynaecological malignancies [6]. Similarly, expression of TDO in cancer cells may promote tumour progression in melanoma, glioma, gynaecological malignancies, lung cancer, renal and bladder cancers (reviewed in [5]). Moreover, the contribution of selected tryptophan metabolites including kynurenine (KYN), quinolinic acid (QUIN) and 3-hydroxyanthranilic acid (3-HAA) in the progression of tumours has been previously presented (reviewed in [4]). However, the potential role in the process of carcinogenesis of another endogenous metabolite of tryptophan–kynurenic acid (KYNA) has not been fully reviewed.

KYNA is produced enzymatically from the key intermediate in the tryptophan catabolic pathway—KYN [7, 8]. Initial studies concerning the biological role of KYNA in the central nervous system revealed its neuroprotective and anticonvulsant properties based on receptor interactions [9–13]. Recent studies also underlined the potential role of KYNA in cognitive and memory impairments [14, 15]. However, KYNA is endogenously produced by several types of cells and tissues (brain tissue [16], retina [17]), and was detected in physiological fluids including urine, serum, amniotic fluid [18], milk [19], cerebrospinal fluid [16], synovial fluid [20], saliva [21], gastric juice, bile, pancreatic juice [22], mucus of rat small intestine [23], mucus of pig colon [22] and mucus aspirated from the human caecum or colon ascendens [24]. Importantly, KYNA was also found in several products of the daily human diet and medicinal herbs [25, 26]. The highest concentration was reported in honeybee products and some fresh vegetables, including broccoli and potato [25]. Furthermore, previous studies have indicated that KYNA is absorbed from the intestine into the blood stream and reaches high concentrations in the liver and kidneys [25]. Even though the role of KYNA in physiological and pathological processes in the brain has been excessively studied, its importance on the periphery is still not fully elucidated. Previous studies indicated anti-inflammatory [27, 28], analgesic [29], antiulcerative [30–32], antiatherogenic [33], antioxidative [34] and hepatoprotective [35] properties of KYNA. More recent studies are focused on the potential role of KYNA in carcinogenesis and therapy, however, the results are ambiguous and the biological activity of KYNA in these processes has not been unequivocally established.

## KYNA content in cancer tissue and body fluids

There are several studies focused on the KYNA content in various types of cancer (Table 1), which revealed some differences in KYNA concentration between cancer and physiological conditions. It was confirmed that KYNA is present in the colon adenocarcinoma [36], glioblastoma [37], renal cell carcinoma (RCC) [38] and oral squamous cell carcinoma (SCC) [39] tumour tissue. The highest KYNA concentration was reported in the colon adenocarcinoma [36], and a comparable amount of KYNA was detected in glioblastoma [37] and RCC [38] tumours, but KYNA content in oral SCC tissue was significantly lower [39]. Interestingly, there are ambiguous data concerning the differences in KYNA content in tumour and healthy tissue. KYNA concentration was considerably higher in the colon adenocarcinoma and oral SCC than in healthy colon mucosa [36] and oral mucosa [39]. On the other hand, there are some data presenting an inverted relationship. Walczak et al. [38] reported that KYNA concentration was considerably lower in RCC than in healthy renal tissue (Table 1).

**Table 1:** Content of KYNA in tumour tissue, blood and urine from patients with cancer

Cancer/type	Tissue	KYNA content		References
		Cancer	Equivalent healthy tissue/fluid	
Glioblastoma	Tumour	100.3 pmol/g wet weight ~ 100.3 nM^c^	–	[37]
Colon adenocarcinoma	Tumour	169.39 pmol/g wet weight ~ 169.39 nM^c^	80.13 pmol/g wet weight ~ 80.13 nM^c^	[36]
Renal cell carcinoma (RCC)	Tumour	115.5 pmol/g wet weight ~ 115.5 nM^c^	379.7 pmol/g wet weight ~ 379.7 nM^c^	[38]
Oral squamous cell carcinoma (SCC)	Tumour	15.85 nM	12.75 nM	[39]
Colon adenocarcinoma	Serum	37.52 nM	26.44 nM	[36]
Lung cancer, adenocarcinoma	Serum	107.1 nM	–	[43]
Lung cancer, squamous cell	Serum	82.1 nM	–	[43]
Lung cancer, large cell	Serum	86.2 nM	–	[43]
Lung cancer, mixed and undifferentiated	Serum	69.5 nM	–	[43]
NSCLC	Serum	93.60 nM	31.40 nM	[45]
Primary cervical cancer	Serum	250 nM	550 nM	[42]
Myelodysplastic syndrome (MDS)	Serum	No data—serum KYNA level higher compared with healthy control	–	[44]
Monoclonal gammopathy of undetermined significance (MGUS) (premalignant condition)	Peripheral blood plasma	87.42 nM	31.58 nM	[41]
Multiple myeloma (MM)	Peripheral blood plasma	59.23 nM	31.58 nM	[41]
Glioblastoma (GBM)	Plasma	21.3 nM	52.89 nM	[40]
Monoclonal gammopathy of undetermined significance (MGUS) (premalignant condition)	Bone marrow plasma	78.41 nM	35.62 nM	[41]
Multiple myeloma (MM)	Bone marrow plasma	53.92 nM	35.62 nM	[41]
Bladder cancer	Urine	Higher excretion of kynurenic acid in males	–	[46]
Bladder cancer	Urine	No difference	14.1 µmol/24 h	[48]
Bladder cancer	Urine	14.13 µmol/24 h (grades 1–2); 12.53 µmol/24 h (grades 3–4)	–	[47]
Prostate cancer	Urine	29.9 mg/L* ~ 158 µM	41.6 mg/L* ~ 220 µM	[53]
Breast cancer, ‘normal breast cancer’^a^	Urine	11.6 µmol/24 h	12.9 µmol/24 h	[50]
Breast cancer, ‘abnormal breast cancer’^a^	Urine	13.5 µmol/24 h	12.9 µmol/24 h	[50]
Breast cancer, ‘without new sites’^b^	Urine	11.0 µmol/24 h	–	[47]
Breast cancer, ‘with new sites’^b^	Urine	11.55 µmol/24 h	–	[47]
Human colon carcinoma	Mucus from caecum or colon ascendens	269.40 nM	82.22 nM	[24]
Adenoma tubulovillosum	Mucus from caecum or colon ascendens	200.50 nM	82.22 nM	[24]
Adenoma tubulare	Mucus from caecum or colon ascendens	243.50 nM	82.22 nM	[24]

*Statistically insignificant

^a^Breast cancer patients were divided into two groups: ‘abnormal breast cancer patients’—patients demonstrating increased level of two or more tryptophan metabolites in comparison to control and ‘normal breast cancer patients’—patients demonstrating increased level of no or one tryptophan metabolite in comparison to control

^b^Breast cancer patients were divided into two groups: ‘with new sites’—patients with conclusive evidence of cancer at new sites, e.g., lung, ovaries, brain and bone and ‘without new sites’—patients without evidence of cancer at new sites

^c^Assuming 1 g wet weight ~ 1 mL

KYNA was detected in the serum of patients diagnosed with cancer in concentrations that ranged from 21.3 to 250 nM depending on the type of cancer [36, 40–44]. Higher KYNA concentration in serum in comparison to healthy population was observed in blood disorders, such as monoclonal gammopathy of undetermined significance (MGUS), representing premalignant conditions [41], multiple myeloma (MM) [41] and myelodysplastic syndrome (MDS) [44]. An elevated KYNA concentration was also detected in the serum of patients diagnosed with colon adenocarcinoma [36]. Interestingly, an elevated serum KYNA level was also associated with the invasiveness of lung cancer. Lung adenocarcinoma is considered as more aggressive than other types of non-small cell lung cancer. Sagan et al. [43] reported that the KYNA level in the serum of patients with lung adenocarcinoma was significantly higher than in lung SCC. Moreover, further studies indicated that circulating KYNA level was higher in non-small cell lung carcinoma (NSCLC) patients than in controls and its concentration level correlated with the stage of cancer progression since nodal involvement is considered as a negative prognostic factor (Table 1). The authors suggested a potent role of KYNA as a marker for non-invasive discrimination between N0 and N+ patients in NSCLC [45].

Additionally, an increased KYNA concentration was observed in bone marrow plasma of MGUS and MM patients [41]. Interestingly, the KYNA level in the bone marrow plasma of MGUS patients was significantly higher than in the MM group [41] (Table 1).

On the other hand, Fotopoulou et al. [42] observed in patients with primary cervical cancer a more than twofold decrease of serum KYNA concentration in comparison to a control (healthy) group. A similar effect was noted for patients diagnosed with glioblastoma [40]. KYNA concentration in plasma from glioblastoma patients was significantly lower than in plasma from healthy volunteers (Table 1) [40]. However, the reason for this phenomenon has not been clarified. Taking into consideration the increase in the activation of the kynurenine pathway (KYN/TRP ratio) along with a decrease in the concentration of neuroactive metabolites including KYNA in glioblastoma patient plasma compared to controls [40], it cannot be excluded that cancer cells change the route of kynurenine pathway to produce more effectively NAD^+^ as a source of energy for their excessive metabolic processes.

There are a few studies concerning the potential influence of cancer on KYNA urinary excretion, as this tryptophan metabolite is mainly excreted from the body with urine. Fujinaga et al. [46] reported increased KYNA urinary excretion in a group of males diagnosed with bladder cancer. However, most studies did not confirm any relationship between KYNA excretion and bladder or kidney cancer [47, 48]. Additionally, no correlation between bladder cancer grade and KYNA content in urine was found [47]. Early studies also excluded the potential role of KYNA in the process of carcinogenesis in mouse bladder [49]. Similarly, Davis et al. [50] did not report any differences in KYNA urinary excretion between breast cancer patients and healthy subjects. On the other hand, there are some data suggesting that elevated KYNA urinary excretion after l-tryptophan loading may be considered as a potential diagnostic marker for some types of cancer [46, 47, 50–52]. Gailani et al. [51] reported increased urinary excretion of KYNA in 25% of patients diagnosed with bladder cancer after alimentary administration of l-tryptophan. These results were confirmed in the later studies by Fujinaga et al. [46]. KYNA level after l-tryptophan loading was also elevated in urine of breast cancer patients, demonstrating increased level of two or more tryptophan metabolites in comparison to control [50]. Although there was no correlation between breast cancer stage and basal KYNA urinary excretion, breast cancer patients with conclusive evidence of cancer at new sites, e.g., lung, ovaries, brain and bone, were characterized by elevated KYNA excretion after l-tryptophan administration [47]. Additionally, Gailani et al. [52] reported an increased urinary excretion of KYNA in 18% of patients diagnosed with Hodgkin’s disease 24 h after administration of l-tryptophan. However, a similar increase in KYNA excretion was observed in only 1 of 18 patients with lymphosarcoma enrolled to the study [52].

One of the recent studies carried by Gkotsos et al. revealed some changes in KYNA concentration in urine in the group of patients diagnosed with prostate cancer, but the authors finally concluded that KYNA cannot be considered a reliable marker for monitoring the progress of the disease [53]. The median of KYNA concentration was decreased in samples obtained from patients with prostate cancer in comparison to control group by 28%, however, these differences are not statistically significant. Moreover, there was no correlation between KYNA concentration in urine and the cancer Gleason grade and the age of patients [53]. On the other hand, measurements of KYNA concentration in urine may improve detection of prostate cancer in patients undergoing prostatic massage procedure. KYNA concentration in urine was higher before prostatic massage (Table 1) [53].

Studies on the content of KYNA in the human colon revealed a considerably higher KYNA concentration in mucus samples aspirated from the human caecum or colon ascendens from patients diagnosed with colon carcinoma, *Adenoma tubulovillosum* or *Adenoma tubulare* than in a healthy control group (Table 1) [24]. In this regard, KYNA concentration in mucus obtained from patients with a benign polypoid lesion Polipus hyperplasticus did not differ significantly from the control level [24].

**Fig. 2:**
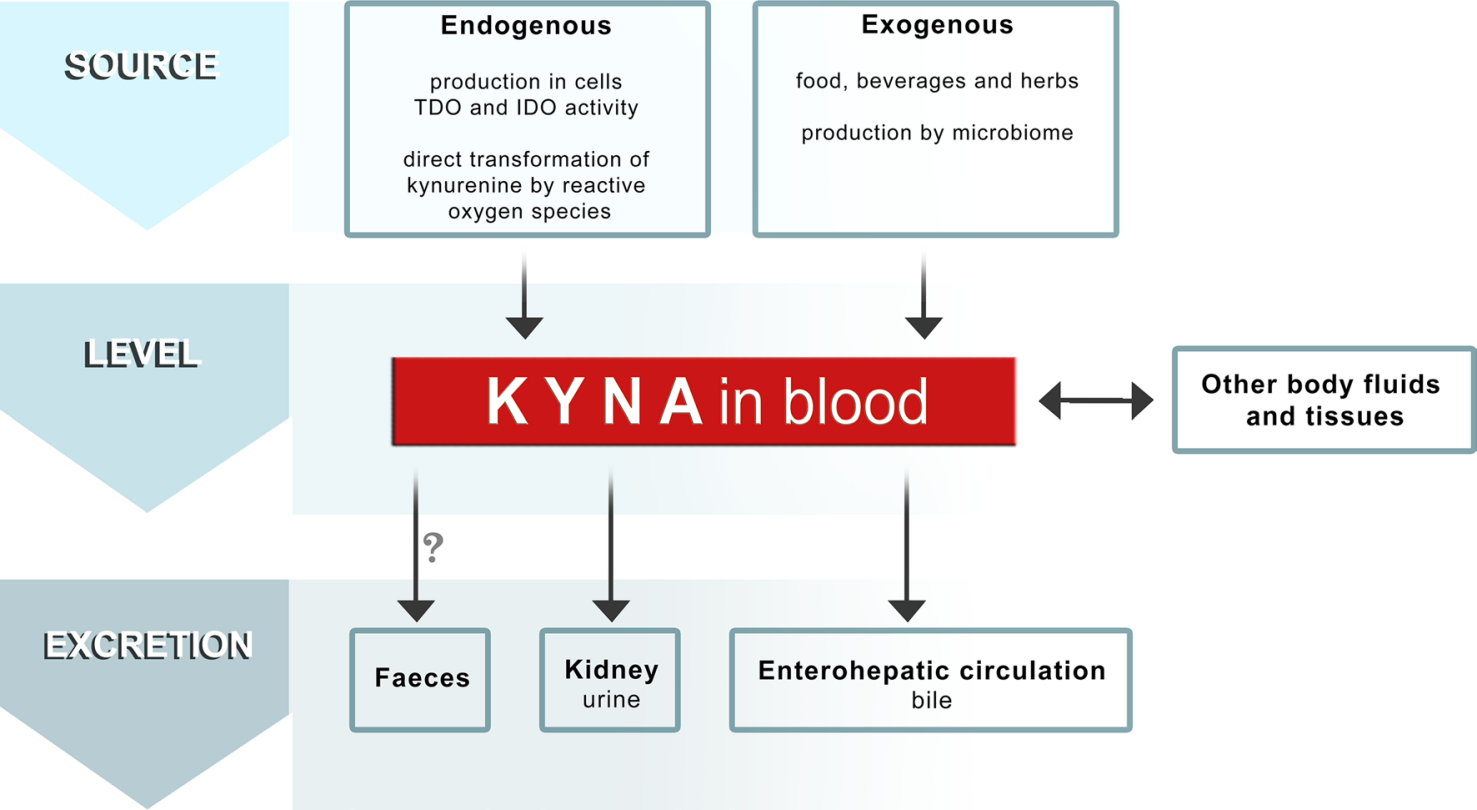
The fate of KYNA in the body—a schematic representation of factors affecting its level. The level of KYNA in the tissues and body fluids depends on its endogenous production, supply and elimination from the body. KYNA is endogenously produced in cells and as a result of indoleamine 2,3-dioxygenase (IDO) and tryptophan 2,3-dioxygenase (TDO) activity [2, 3, 24] or by direct transformation of kynurenine to KYNA by reactive oxygen species [126]. KYNA is also delivered to the body with food, beverages and herbs [25, 57, 118]. Importantly, intestinal microflora may participate in the formation of the overall amount of KYNA in gastrointestinal tract [23]. KYNA concentration in serum may be also dependent on the efficiency of KYNA excretion from the body with urine. However, it was suggested that KYNA may be excreted with bile as a consequence of the enterohepatic circulation [22]. Our unpublished data showed that KYNA is present in faeces, but its origin (undigested food, intestinal microflora or the way of KYNA excretion from the body) has not been elucidated. Question mark (?) means that there is no direct evidence that KYNA found in faeces comes from blood

Summarizing previously published results indicating the presence of KYNA in cancer tissues and body fluids of subjects with cancer, and differences of KYNA concentration between cancer patients and healthy control group, some of the critical points are worth to be mentioned. The level of KYNA in the tissues and body fluids depends on its endogenous production, supply and elimination from the body (Fig. 2). Therefore, KYNA concentration in serum and urine might not exclusively reflect the ongoing cancer process in the body. KYNA is produced endogenously by normal and cancer cells [24, 40, 41, 54, 55], however, exogenous sources of this compound should not be ignored. KYNA is present in several beverages and products of daily diet. Importantly, KYNA may be absorbed from the gastrointestinal tract and transported with blood stream to peripheral organs [25, 56, 57]. Moreover, previous studies revealed that intestinal microflora may participate in the formation of the overall amount of KYNA in gastrointestinal tract [23]. In turn, KYNA itself may affect bacterial growth and thus, the biodiversity composition of microbiome [58, 59]. Unfortunately, none of the above-mentioned studies concerning KYNA concentration in serum and other body fluids considers the potential effect of exogenous sources of KYNA on the final results. On the other hand, KYNA concentration in serum may be also dependent on the efficiency of KYNA excretion from the body. In fact, previous studies revealed increased KYNA concentration in serum of patients with renal failure [60, 61]. Since it was suggested that KYNA may be excreted with bile as a consequence of the enterohepatic circulation [22] it can be speculatively suggested that disruption of this secretion–absorption cycle may significantly influence the KYNA content. Thus, it seems to be reasonable to postulate that pharmacokinetics of KYNA should be carefully considered in further studies devoted to determine cause and effect relationships between KYNA content and cancer promotion and progression.

Taking into consideration the potential influence of endogenous and exogenous sources of KYNA and the process of its excretion on the overall KYNA level in physiological fluids, measurements of this compound in cancer tissue and its surrounding seem to be the most relevant. The majority of studies revealed the elevated KYNA level in tumours in comparison to healthy tissue [36, 39]. However, decreased concentration of KYNA in RCC might be associated with loss of filtration properties by cancer tissue [38]. Importantly, the possible relationship between KYNA content and type of biopsy specimen has not been investigated so far. The critical differences in KYNA content in necrotic core of solid tumours and highly proliferating surface layers cannot be excluded.

## KYNA production in cancer cells in vitro

In vitro experiments confirmed that KYNA is produced by both normal and cancer cells (Table 2). Taking into consideration the significant role of KYNA in the central nervous system, it is not surprising that the first in vitro studies on KYNA production in cancer cells were conducted with glioma. Vezzani et al. [54] reported de novo synthesis of KYNA in slices of human gliomas obtained from biopsy material. Importantly, astrocytomas produced considerably more KYNA than glioblastomas. Similarly, the efficient synthesis of KYNA was confirmed in rat glioma C6 cells [55]. Moreover, Adams et al. [40] revealed that the expression of kynurenine aminotransferase I (KAT I), one of the key enzymes in KYNA synthesis, was significantly decreased after stimulation with interferon-gamma (IFN-γ) in human glioma cells, which significantly lowered the KYNA/KYN neuroprotective ratio.

**Table 2:** KYNA production in cancer cells in vitro

Cell type	Cell line	KYNA production	Comments	References
Human gliomas	Biopsy material	43.7 ± 12.2 pmol KYNA/mg protein/2 h	*Astrocytoma* (incubation with 200 μM l-KYN, 2 h)	[54]
		10.4 ± 2.5 pmol KYNA/mg protein/2 h	*Glioblastoma* (incubation with 200 μM l-KYN, 2 h)	
Rat glioma	C6	29.7 pmol KYNA/mg protein/2 h	Incubation with 5 µM l-KYN, 5 h	[55]
Human myeloma	U266	~ 7 pmol KYNA/5 × 10^5^ cells/2 h	Incubation with 5 µM l-KYN, 2 h^a^	[41]
	RPMI8226	~ 1.5 pmol KYNA/5 × 10^5^ cells/2 h		
Human colon adenocarcinoma	Caco-2	4.21 pmol/1 × 10^5^ cells/2 h	Incubation with 5 µM l-KYN, 2 h	[24]
	HT-29	1.39 pmol/1 × 10^5^ cells/2 h		
	LS-180	1.18 pmol/1 × 10^5^ cells/2 h		

^a^Estimated from graphs

Zdzisińska et al. [41] reported KYNA production in the bone marrow stromal cells (BMSCs) of control and MM patients and by two myeloma cell lines (U266, RPMI8226). However, no significant differences in KYNA production in BMSCs of healthy control subjects and MM patients were observed. Moreover, KAT I and KAT II were expressed in BMSCs of healthy control subjects, as well as in BMSCs of patients diagnosed with MM and MGUS. Interestingly, despite the physiological similarity between examined myeloma cell lines, their ability to produce KYNA differed significantly. KYNA production in U266 cells was almost fivefold higher than in RPMI8226 cells [41].

Interesting results were obtained from the studies on KYNA production in colon epithelial and cancer cells. KYNA synthesis in colon adenocarcinoma cell lines Caco-2, HT-29 and LS-180 was considerably higher in comparison to normal colon epithelial cells [24].

## Effect of KYNA on cancer cell proliferation in vitro

Taking into consideration KYNA production by cancer cells, the principal question is whether it may play any role in the process of carcinogenesis or cancer progression. There are only a few studies concerning the effect of KYNA on cancer cells’ proliferation, thus, this matter has not been fully elucidated (Table 3). Di Serio et al. [62] reported a major stimulatory effect on the proliferation rate of mouse microglia N11 (ED_50_ = 25 nM) and human glioblastoma U-343 MG cells. This effect was not observed in the mouse (J774) and human (U937) macrophage cell lines. Importantly, the mentioned study was conducted with the use of reduced serum culture medium (supplemented with 3% serum) [62], and the stimulatory effect of KYNA on cancer cell proliferation has not been confirmed under normal experimental conditions (10% serum) in other types of cancer cells [24, 37, 38, 63]. It should be noted that serum deprivation itself inhibited proliferation of U-343 MG cells in comparison to standard conditions and importantly, KYNA did not fully reverse this effect [62]. However, it cannot be excluded that KYNA interacts with different elements of signalling pathways or cell cycle regulators under stress conditions. Interestingly, cultured human glioma cells exposed to INF-γ presented significantly lower expression of KAT I [40]. It cannot be excluded that immune system may play an important role in biological response of cancer cells exposed to KYNA.

**Table 3:** Effect of KYNA on proliferation, DNA synthesis and migration of cancer cells in vitro

Cellular process/detection method	Cell type	Cell line	Effect	Comments	References
Proliferation	Human glioblastoma	U-343 MG	Increase in growth rate	KYNA 1 µM and 10 µM; medium containing low serum (3%)	[62]
	Human glioblastoma	T98G	Antiproliferative effect	IC_50_ = 1.3 mM	[37]
	Human colon adenocarcinoma	Caco-2		IC_50_ = 1.2 mM	[24]
		HT-29		IC_50_ = 0.9 mM	
		LS-180		IC_50_ = 0.2 mM	
	Human renal cell carcinoma	Caki-2		IC_50_ = 0.04 mM	[38]
DNA synthesis (BrdU)	Human glioblastoma	T98G	Decreased DNA synthesis	IC_50_ = 8.9 mM	[37]
	Human colon adenocarcinoma	HT-29		IC_50_ = 4.4 mM	[63]
	Human renal cell carcinoma	Caki-2		IC_50_ = 2.13 mM	[38]
Migration (wound assay)	Human glioblastoma	T98G	Inhibition of migration	IC_50_ = 0.6 mM	[37]
	Human renal cell carcinoma	Caki-2		47% in comparison to control after 24 h of incubation with KYNA 2.5 mM	[38]

On the contrary, recent studies revealed an inhibitory effect of KYNA on the proliferation of human glioblastoma T98G cells (IC_50_ = 1.3 mM) [37]. It also significantly inhibited the proliferation of human colon and renal cancer cells [24, 38]. KYNA exerted an antiproliferative activity against colon adenocarcinoma HT-29, LS-180 and Caco-2 cells with IC_50_ of 0.9, 0.2 and 1.2 mM, respectively [24]. Importantly, it should be indicated that, a clear-cut dose-dependent inhibitory effect was observed in concentrations above 0.01 mM for HT-29 cells [24]. In vitro studies demonstrated antiproliferative properties of KYNA also against RCC Caki-2 cells (IC_50_ = 0.04 mM) [38].

**Fig. 3:**
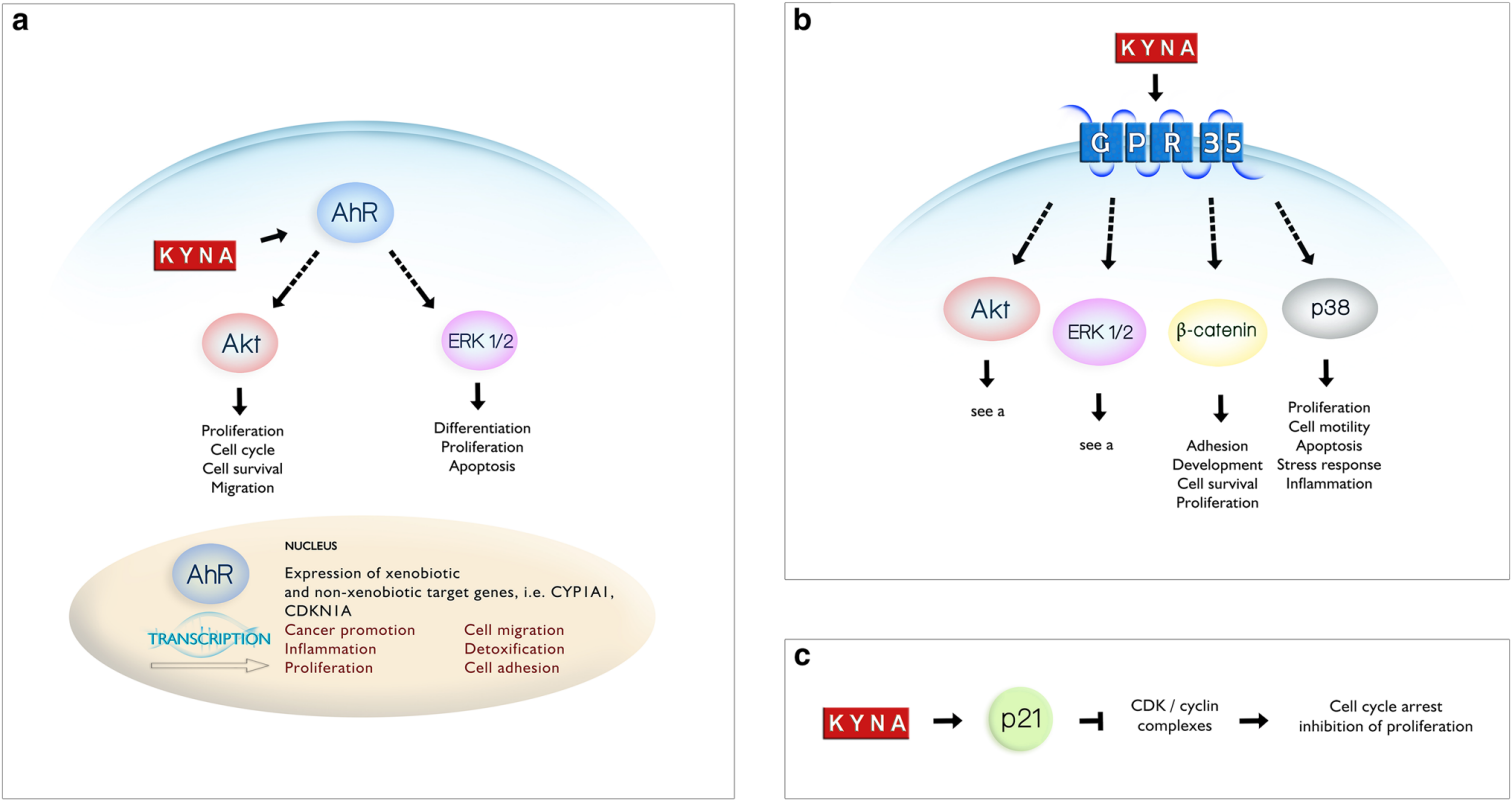
A schematic presentation of selected cellular processes influenced by KYNA. KYNA inhibits phosphorylation of protein kinase B (Akt), extracellular signal-regulated kinase (ERK 1/2), and p38 mitogen-activated protein kinase (p38) [99]. The effect of KYNA on phosphoinositide 3-kinase/protein kinase B PI3K/Akt pathway may lead to disruption of various cellular processes including proliferation, cell cycle, cell survival and migration. Interaction between KYNA and ERK pathway may affect the processes of differentiation, proliferation or apoptosis, whereas interaction between KYNA and p38 pathway may affect the processes of proliferation, cell motility, apoptosis, stress response and inflammation. Moreover, KYNA enhances the protein expression of β-catenin which is involved in adhesion, development, cell survival and proliferation. The potential relationship between AhR (**a**) and GPR35 (**b**) receptors and mentioned signalling pathways is presented; however, the specific mechanism of KYNA interactions has not been fully elucidated. **a** KYNA is an agonist of the aryl hydrocarbon receptor AhR. According to the biological effects of AhR activation, three possible types of interactions may be suggested: via PI3K/Akt pathway, ERK signalling pathway or by activation of the expression of xenobiotic and non-xenobiotic target genes (based on [127–129]). **b** KYNA activates G-protein-coupled receptor 35 (GPR35) which may lead to inhibition of phosphorylation of various signalling proteins, including Akt, ERK, and p38 mitogen-activated protein kinase (p38). Additionally, activation of GPR35 may lead to increase in β-catenin expression (based on [59, 130]). **c** KYNA enhances the protein expression of cyclin-dependent kinase (CDK) inhibitor p21^Waf1/Cip1^ resulting in possible inhibition of cyclin D/CDK4/CDK6, cyclin E/CDK2, cyclin A/CDK2 and cyclin B/CDK1 complexes which may lead to cell cycle arrest and antiproliferative activity [63]

Importantly, KYNA inhibited other cellular processes in cancer cells. KYNA in millimolar concentrations significantly decreased DNA synthesis in T98G (IC_50_ = 8.9 mM) [37], HT-29 (IC_50_ = 4.4 mM) [63] and Caki-2 (IC_50_ = 2.1 mM) [38] cells. It also inhibited the migration of glioblastoma (IC_50_ = 0.6 mM) [37] and RCC [38] cells. Although the exact molecular mechanism of the action of KYNA on cancer cells’ proliferation and motility has not been revealed, its possible interactions with receptors and several proteins involved in signal transmission and cell cycle regulation suggest the potential function of KYNA in the process of carcinogenesis (Fig. 3).

## Effect of KYNA on receptors and intracellular processes in cancer cells

### Receptors

Despite the fact that KYNA was initially found to block all ionotropic glutamate receptors [64], later studies indicated its higher potency as a competitive antagonist of the strychnine-insensitive glycine co-agonist site of the *N*-methyl-d-aspartate (NMDA) receptor [65] and as a non-competitive inhibitor of the α-7 nicotinic acetylcholine receptor (alpha7 nAChR) [66]. Moreover, KYNA is an agonist of G-protein coupled receptor (GPR35), which is predominantly expressed in immune and gastrointestinal tissues [67], and an agonist of aryl hydrocarbon receptor (AhR) [68, 69]. Thus, KYNA may exert various biological effects on cancer cells by interaction with specific receptors (Table 4).

**Table 4:** Effect of KYNA on cellular processes

Cellular process	Agent/factor	Cell type	Effect/comments	References
Receptor interaction	Glutamate receptors (NMDA, AMPA, kainate)	Hippocampal and cerebral cortical neurons, baby rat hemisected spinal cord	Antagonist	[64–66]
	Alpha7 nicotinic receptor	Hippocampal neurons in cultures and in slices	Antagonist	[66]
	GPR35	Chinese hamster ovary (CHO) cells transfected with human GPR35	Agonist	[67]
	AhR	Human liver hepatocellular carcinoma (HepG2)	Agonist	[68]
		Rat basophilic leukaemia cell (RBL-2H3)		[69]
Transporter interaction	Multidrug resistance protein 4 (MRP4)	Human embryonic kidney cells (HEK293)—MRP4 over-expressing membrane vesicles; 0.1 mM KYNA	MRP4 substrate	[113]
	Multidrug resistance protein 4 (MRP4)	Human embryonic kidney (HEK293) transduced with baculoviruses of human MRP4; membrane vesicle preparation	KYNA inhibited substrate-specific uptake by MRP4 in a concentration range of 0.1–1 mM	[114]
	Breast cancer resistance protein (BCRP)	Human embryonic kidney (HEK293) transduced with baculoviruses of human BCRP; membrane vesicle preparation	KYNA inhibited substrate-specific uptake by BCRP in a concentration range of 0.1–1 mM	
Cell cycle regulation	p21^Waf1/Cip1^	Human renal cell carcinoma Caki-2	Gradually increased protein expression of p21^Waf1/Cip1^ (15 min–48 h of exposure to KYNA 2.5 mM)	[38]
		Human colon adenocarcinoma HT-29	KYNA 0.01–5 mM, dose-dependent increase of p21^Waf1/Cip1^ (4, 24 and 48 h exposure); no antiproliferative properties of KYNA in cells with silenced p21^Waf1/Cip1^ gene	[63]
	pRb	Human renal cell carcinoma Caki-2	Decreased phosphorylation (24 and 48 h exposure to KYNA 2.5 mM)	[38]
Signal transmission	p38 MAPK	Human renal cell carcinoma Caki-2	Decreased phosphorylation (15 min–48 h of exposure to KYNA 2.5 mM)	[38]
		Human colon adenocarcinoma HT-29	Gradually decreased phosphorylation (24 h exposure to KYNA 0.01–5 mM; 48 h exposure to KYNA 0.1–5 mM)	[99]
	ERK1/2	Human colon adenocarcinoma HT-29	Decreased phosphorylation (4 h exposure to KYNA 5 mM; 24 h exposure to KYNA 0.01–5 mM)	[99]
	Akt	Human colon adenocarcinoma HT-29	Decreased phosphorylation (4 h exposure to KYNA 1–5 mM; 24 h exposure to KYNA 1–5 mM; 48 h exposure to KYNA 0.1–5 mM)	[99]
	β-catenin	Human colon adenocarcinoma HT-29	Increased expression after 24 and 48 h exposure to KYNA 5 mM	[99]

Although the molecular mechanism of antiproliferative activity of KYNA is not fully elucidated, an involvement of glutamate receptors should be considered. Previous studies indicated that glutamate antagonists inhibit proliferation and increased cell death of several cancer cell lines expressing this type of receptors on their surface, including HT-29 cells [70, 71]. Moreover, KYNA reversed the stimulatory effect of glutamate on glioma T98G cell proliferation, and enhanced the antiproliferative effect of glutamate receptor antagonists MK801 and GYKI 52466 [37].

The anticancer potential of KYNA may be also determined by interaction with alpha7 nAChR which is expressed in neuronal and various non-neuronal cells including endothelial and smooth muscle cells [72]. alpha7 nAChR is strongly associated with pro-angiogenic and pro-proliferative activity of nicotine, mediated by ERK signalling pathway [73]. Tu et al. [74] also reported the critical role of this receptor in progression and metastasis of gastric cancer. Thus, it cannot be excluded that antiproliferative activity of KYNA on various cancer cells is mediated, even partially, by the inhibition of alpha7 nAChR. Although the antiangiogenic potential of KYNA, as an antagonist of alpha7 nAChR, in cancer cells has not been excessively studied, Arias et al. [73] reviewed that selective (alpha-bungarotoxin and methyllycaconitine) and non-specific (mecamylamine) antagonists of this receptors inhibited proliferation of endothelial cells and angiogenesis. Importantly, alpha7 nAChR is also involved in the modulation of anti-inflammatory response [75], thus, it is regarded as an attractive target for anticancer therapy.

On the other hand, KYNA is an endogenous agonist of AhR which is involved in several processes including cell proliferation, apoptosis, adipose differentiation, tumour suppression and immune cell differentiation (reviewed in [76]) (Fig. 3a). KYNA in nanomolar concentrations is an efficient agonist for the human AhR inducing xenobiotic metabolism in cells and production of interleukin-6 (IL6), an important mediator of pro-tumorigenic properties [68]. DiNatale et al. [68] revealed that KYNA induces mRNA expression of* CYP1A1* and* CYP1A*-mediated metabolism in vitro.

The potential role of AhR and its ligands in the process of carcinogenesis has been previously reviewed [77]. It was reported that AhR is overexpressed in some cancers including lung carcinoma, gastric carcinoma and medulloblastoma [78] and an oncogenic potential of AhR was reported in hepatocarcinoma and stomach tumour [79]. Most of the data point to a direct and indirect interaction between the AhR receptor and tumour promotion, progression and the phenomenon of immune escape in tumours [80]. Importantly, previous studies suggested the indirect role of AhR signalling in cancer promotion, progression and metastasis by affecting kynurenine pathway and immune response. The expression of some enzymes of kynurenine pathway, IDO1 and TDO2, is strictly controlled by AhR. Activity of IDO1 and TDO2, decreasing the availability of tryptophan in the tumour environment, may lead to suppression of immune response via inhibition of antigen-specific T-cell response and promotion of tolerogenic phenotype in dendritic cells [81, 82]. Thus, potential pro-carcinogenic effect of KYNA as AhR ligand cannot be excluded. However, activation of AhR may also lead to inhibition of the proliferation of various cancer cells, including liver, prostate and breast cancer and intestinal carcinogenesis in mice [83–86]. It was hypothesized that this apparently mutually exclusive function of AhR in tumour progression may be partially dependent on the specific role of various cell types in the process of migration [87]. Although KYNA is considered as an agonist [68], in some cases, its biological impact on cellular processes is similar to the biological activity of AhR antagonist, such as resveratrol [69]. Mutz et al. [88] reported that EWS-FLI1 fusion protein, involved in progression of an aggressive paediatric tumour Ewing sarcoma (ES), suppresses autocrine AhR signalling by interaction with kynurenine pathway. It should be noted that KYNA may also affect immune response via AhR, since this receptor has an important regulatory role in inflammation and its activation prevents proinflammatory cytokine induction in cells exposed to an inflammatory stimulus [89–92].

Despite the fact, that GPR35 agonists have not been directly related to pro- or antiproliferative activity towards cancer cells to date, GPR35 signalling via ERK kinase is involved in several processes including proliferation, cell survival and metastasis [93] (Fig. 3b). Thus, the interaction between KYNA and GPR35 in cancer cannot be excluded. It should be noted that KYNA as an agonist may also modulate the immune response to the initiation and progression of carcinogenesis via direct interaction with GPR35, which is predominantly expressed on immune cells [67]. However, the role exerted by KYNA on the defensive reaction in cancer via GPR35 receptors located on immune cells [67] is as yet unknown. Activation of GPR35 by KYNA induces the internalization of the receptor and leads to calcium mobilisation and production of inositol phosphate (IP_3_) [67].

The direct interaction of KYNA with receptor seems to be a critical step determining biological activity of the substance. However, the involvement of human organic anion transporters 1 (hOAT1) and 3 (hOAT3) in the uptake of KYNA has been also suggested [94]. The mentioned transporters are mainly expressed in the brain and the kidney. Similarly, mining the TCGA [95] and GTEx [96] datasets revealed no or very low expression of genes coding hOAT1 (*SLC22A6*) and hOAT3 (*SLC22A8*) in most cancer types except for brain and renal tumours (data not shown). Importantly, hOAT1 and hOAT3 expression was markedly decreased in renal cancer tissue in comparison to healthy renal tissue. On the other hand, KYNA concentration might be also increased in the cells due to its intracellular synthesis from kynurenine which is taken up by Na^+^-independent transporter of neutral amino acids as shown by Speciale et al. [97] in astrocytes.

### Signalling pathways

Importantly, KYNA has a modulatory effect on signalling pathways which are well-known targets of GPR signalling, including the phosphoinositide 3-kinase (PI3K)/protein kinase B (Akt) and mitogen-activated protein kinase (MAPK) pathways [98, 99]. KYNA was shown as a potent inhibitor of extracellular signal-regulated kinases (ERK) 1/2, p38 MAPK, and Akt [99] (Fig. 3). These signalling pathways are directly involved in processes of proliferation, survival, apoptosis and migration [100]. The effect of KYNA on signalling pathways in cancer cells was probed in colon adenocarcinoma [63, 99] and renal cancer cells [38]. Importantly, KYNA in high concentrations exerted an inhibitory effect on signalling pathways in cancer cells, which is consistent with the concentration range at which KYNA inhibits tumour cell proliferation. KYNA in millimolar concentrations decreased phosphorylation of ERK 1/2 in a dose-dependent manner in HT-29 adenocarcinoma cells [99]. It might be suggested that p38 kinase is more sensitive to the inhibitory effect of KYNA, as inhibition of p38 phosphorylation was observed at 10 times lower concentration of KYNA [99]. Importantly, a similar effect of p38 inhibition was observed in renal cell carcinoma Caki-2 cells [38]. Moreover, KYNA in a dose-dependent manner inhibited phosphorylation of Akt in colon adenocarcinoma HT-29 cells, the main element of PI3K/Akt pathway whose disturbance may lead to cancer promotion and progression [99].

On the other hand, KYNA interaction with GPR35 and mentioned signalling pathways may also affect immunological response to cancer cells. It was suggested that KYNA–GPR35 interaction might lead to suppression or limitation of inflammation [59]. Moreover, KYNA leads to β-catenin accumulation in colon cancer cells which may also be dependent on interaction with GPR [99, 101] (Fig. 3). Interestingly, β-catenin (WNT) signalling pathway is involved in inhibition of inflammation via NF-κB pathway [59, 102]. All of these data suggested potential double-edged role of KYNA in carcinogenesis. Taking into consideration an anti-inflammatory potential of KYNA, this compound may be considered as a potent anticancer agent since continuous inflammation is one of the factors which may induce process of carcinogenesis [103, 104]. On the other hand, the proper immune response may inhibit the early stages of carcinogenesis or prevent spreading of cancer cells into the whole body [105, 106].

### Cell cycle regulators

Moreover, recent studies revealed that KYNA affected cell cycle regulators and downstream elements of signalling pathways, which lead to antiproliferative activity towards cancer cells (Table 4). One of the affected proteins is cyclin-dependent kinase inhibitor p21^Waf1/Cip1^ (Fig. 3c). KYNA in colon cancer HT-29 and renal cancer Caki-2 cells significantly enhanced expression of p21^Waf1/Cip1^, which is involved in cell cycle regulation, apoptosis and cell differentiation [38, 63, 107, 108]. Inhibition of phosphorylation of Rb in Caki-2 cells exposed to KYNA was also reported [38]. Importantly, this protein controls progression through the restriction point within the G1 phase of the cell cycle, and thus, regulates cell growth and proliferation. KYNA affects also signalling kinases inhibiting p38 MAPK in Caki-2 cells, which is involved in the control of cell cycle in G1/S and G2/M checkpoints through regulation of various genes coding cytokines, transcriptional factors and receptors [38, 109, 110].

## KYNA and cancer therapy: future perspectives and potential risks

Summarizing all data, the question arises whether KYNA may have some clinical significance. Although there are limited data, the future use of KYNA as an anticancer agent or as a supportive agent in standard cancer therapy cannot be excluded. Undoubtedly, KYNA in millimolar concentrations exerted antiproliferative activity towards several cancer cell lines [24, 37, 38], and its local concentration may be easily increased. Previous studies indicated that it is absorbed from the intestine into the blood stream and reaches high concentrations in the liver and kidney [25]. Moreover, it was reported that the intravenous administration of KYNA in a dose as high as 100 mg/kg/h in rats is well tolerated [111]. A similar effect was observed after long-term application of KYNA in the drinking water [112]. It is also worth mentioning the possible interaction of KYNA with cytostatic drug used in standard chemotherapy. A recent study revealed that KYNA enhanced the antiproliferative effect of temozolomide, a drug used in glioblastoma therapy [37]. Although the molecular mechanism of this phenomenon has not been elucidated, the involvement of glutamate receptors may be suggested. Rzeski et al. [71] showed that glutamate antagonists enhanced the antiproliferative effect of cytostatic drugs, including cyclophosphamide and thiotepa in rhabdomyosarcoma and neuroblastoma cells. However, the other mechanism should be also considered. Previous studies revealed that KYNA inhibited substrate-specific uptake by two important efflux pumps—multidrug resistance protein 4 (MRP4) [113] and breast cancer resistance protein (BCRP) [114], expressed in a broad spectrum of tissues including stem cells, placenta, liver, small intestine, colon, lung, kidney [115]. Despite their important physiological functions in the detoxification of xenobiotics, they are also involved in drug resistance in cancer. KYNA as an inhibitor may prevent this phenomenon, thereby enhancing the effectiveness of standard cytostatic drugs used in cancer therapy. Numerous results consistently indicate that KYNA in high concentrations inhibits viability, proliferation and migration of many cancer cell lines in vitro [24, 37, 38, 63]. Compatible with these findings are reports on the action of KYNA on cell cycle regulators and signalling pathways involved in cell proliferation [38, 63, 99]. Furthermore, recent studies revealed that KYNA inhibits MRP4 [113] and BCRP [114] transporters which are responsible for drug resistance in cancer. Interestingly, it was found that KYNA potentiates effectiveness of the cytostatic drug [37] used in the therapy of cancer. Thus, the use of KYNA as an adjunctive treatment in cancer medication and/or dietary supplementation in cancer prevention cannot be excluded.

**Fig. 4:**
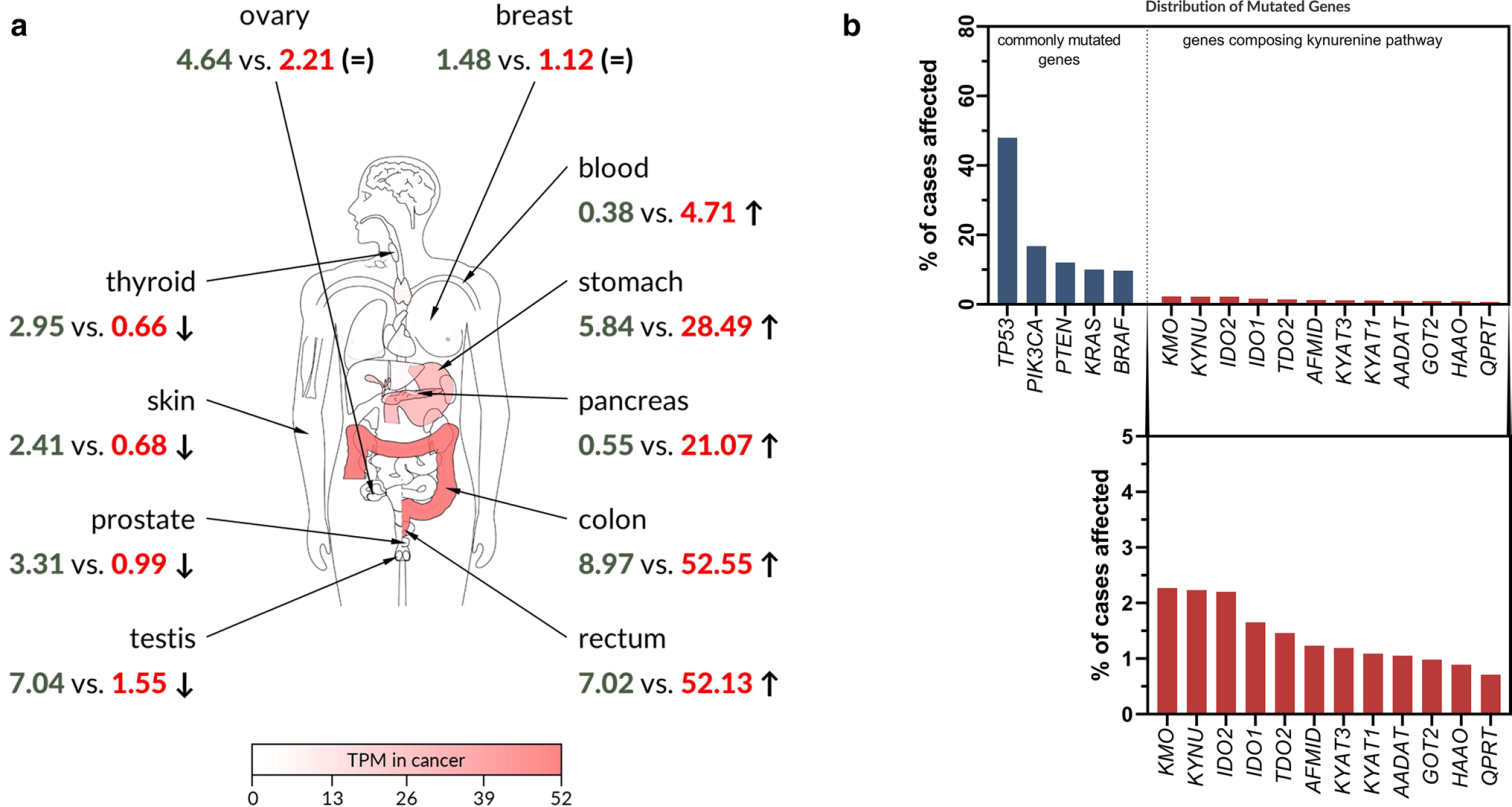
Data mining on *GPR35* gene expression and on mutation frequency of genes composing kynurenine pathway. **a***GPR35* gene expression profiles across normal tissues and paired tumours and were generated using GEPIA2 [131] based on TCGA [95] and GTEx [96] datasets. Retrieved expression values are provided in transcripts per million (TPM) as normal (green) vs. cancer (red) for every listed tissue. Four types of tumours were identified to display significant downregulation of GPR35 (left side of the panel; marked by ↓). Five different types of tumours showed significant upregulation of *GPR35* in comparison to respective normal tissues (right side of the panel; marked by ↑). No significant changes in *GPR35* expression in other types of tumours were identified. Two examples of such tumours are given on the top of the panel (=). Statistical analysis: ANOVA (*q* value cutoff = 0.01;|Log_2_FC| cutoff = 1). Red colour intensity on different parts of the depicted human anatomical outline represents the expression level of GPR35 in human cancers. TPM, transcripts per million; ↑, upregulation of GPR35 in cancer vs. normal; ↓, downregulation of GPR35 in cancer vs. normal; (=), no significant changes in GPR35 expression. **b** Data on the frequency of mutations in genes involved in tryptophan catabolism on the kynurenine pathway were extracted from TCGA [95] database through Genomic Data Commons Data Portal available at https://portal.gdc.cancer.gov. A set of five genes commonly mutated in human cancers were provided for comparison. *TP53* tumor protein p53, *PIK3CA* phosphatidylinositol-4,5-bisphosphate 3-kinase catalytic subunit alpha, *PTEN* phosphatase and tensin homolog, *KRAS* KRAS proto-oncogene, GTPase, *BRAF* B-Raf proto-oncogene, serine/threonine kinase, *KMO* kynurenine 3-monooxygenase, *KYNU* kynureninase, *IDO2* indoleamine 2,3-dioxygenase 2, *IDO1* indoleamine 2,3-dioxygenase 1, *TDO2* tryptophan 2,3-dioxygenase, *AFMID* arylformamidase; *KYAT3* (*KAT* *III*), kynurenine aminotransferase 3, *KYAT1* (*KAT* *I*) kynurenine aminotransferase 1, *AADAT* (*KAT* *II*) aminoadipate aminotransferase, *GOT2* (*KAT* *IV*), glutamic-oxaloacetic transaminase 2, *HAAO* 3-hydroxyanthranilate 3,4-dioxygenase, *QPRT* quinolinate phosphoribosyltransferase

Given our current state of knowledge, types of cancers with the genetic vulnerabilities to KYNA cannot be clearly identified. However, the expression of specific receptors, including AhR, GPR35 and glutamate receptors, in cancer cells increases the probability of KYNA interaction. Interestingly, the expression of GPR35 differs in various cancer types in comparison to normal tissue. All cancer types can be divided into three groups: cancers with decreased expression of GPR35 (i.e., thyroid, prostate and testicular cancer), cancers with GPR35 overexpression (i.e., pancreatic, stomach and colon cancer) and cancers with constant expression of GPR35 (i.e., ovarian and breast cancer) (Fig. 4a), which may be a suggestion for future therapeutic treatment.

**Fig. 5:**
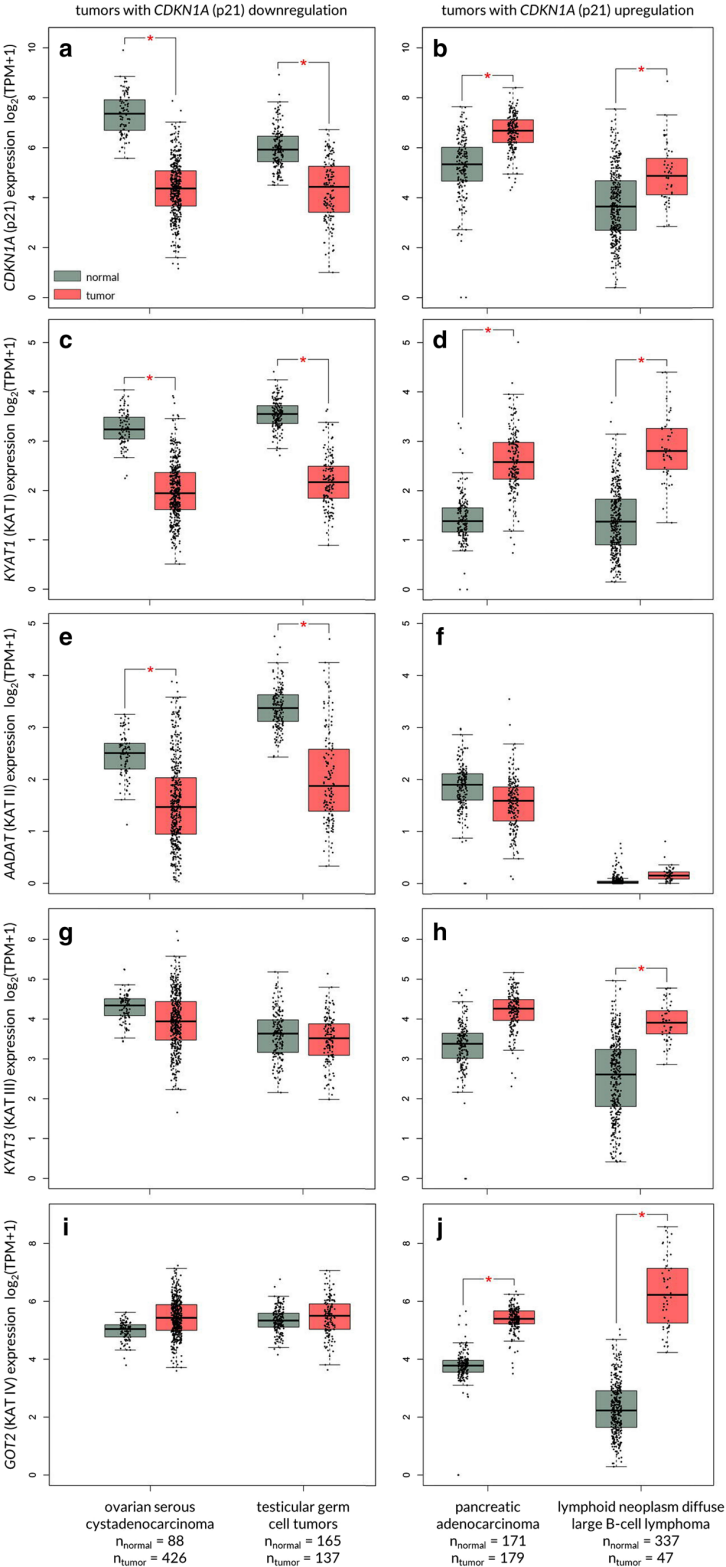
Expression pattern of genes coding kynurenine aminotransferases (KAT I–IV) in human tumours with down- and upregulated CDKN1A (p21^Waf1/Cip1^). GEPIA2 [131] was queried for tumours displaying significant changes in the expression of *CDKN1A*, gene encoding for p21^Waf1/Cip1^ cyclin-dependent kinase inhibitor. Ovarian serous cystadenocarcinoma and testicular germ cell tumour displayed significant downregulation of *CDKN1A* in comparison to paired normal tissues (**a**). On the contrary, pancreatic adenocarcinoma and lymphoid neoplasm diffuse large B-cell lymphoma showed significant upregulation of *CDKN1A* (**b**). Expression of genes coding for kynurenine aminotransferases, i.e., *KYAT1* (KAT I; **c**, **d**), *AADAT* (KAT II; **e**, **f**), *KYAT3* (KAT III; **g**, **h**) and *GOT2* (KAT IV; **i**, **j**), was examined in the same set of cancers. Significant downregulation of *KYAT1* (**c**) and *AADAT* (**e**), but not of *AADAT* (**g**) nor of *GOT2* (**i**), was observed in ovarian serous cystadenocarcinoma and testicular germ cell tumours–tumours displaying *CDKN1A* (p21^Waf1/Cip1^) downregulation. *CDKN1A* (p21^Waf1/Cip1^) upregulation in pancreatic adenocarcinoma was accompanied by an increase in *KYAT1* (KAT I; **d**) and *GOT2* (KAT IV; **j**). Similarly, lymphoid neoplasm diffuse large B-cell lymphoma showed increase in *KYAT1* (KAT I, **d**), *KYAT3* (KAT III; **h**) and *GOT2* (KAT IV; **j**). Differences in the expression levels were analysed by ANOVA. *p < 0.01 and fold-change threshold (|Log_2_FC| cutoff) of 1

Taking into consideration the KYNA-mediated increase of p21^Waf1/Cip1^ protein expression in colon adenocarcinoma cells [63], the other group of tumours probably susceptible to KYNA are tumours with decreased expression of this cell cycle regulator, including ovarian serous cystadenocarcinoma and testicular germ cell tumours (Fig. 5). Importantly, cancers with decreased expression of *CDKN1A* have also decreased expression of genes coding KATs, enzymes directly involved in KYNA synthesis (Fig. 5). Similarly, in pancreatic adenocarcinoma and lymphoid neoplasm diffuse large B-cell lymphoma cells, representing cancers with *CDKN1A* overexpression, the KATs expression is significantly elevated (Fig. 5).

On the other hand, the conclusion that KYNA might promote carcinogenesis may be of great importance. Mutations in genes coding enzymes of the kynurenine pathway in tumour tissue are relatively rare in comparison to the frequency of mutations in *TP53*, *PIK3CA*, *PTEN*, *KRAS* and *BRAF* genes (Fig. 4b). However, in most cases KYNA concentration was increased in cancer tissue and biological fluids in comparison to control [24, 36, 39, 41, 45], suggesting that lowering KYNA level may somehow improve the clinical outcome of cancer patients. However, KYNA deficiency status has not been revealed so far, due to its endogenous production by cells as well as continuous supply with daily diet [21, 25, 56, 57]. On the other hand, the current state of knowledge does not allow to state clearly whether increased concentration of KYNA in tumours is the cause or the effect of the carcinogenesis. Some results point to the pro-carcinogenic properties of KYNA, e.g., enhanced proliferation of certain cell lines in the presence of KYNA [62] and the modulatory action of KYNA on AhR receptors [68, 69], which are believed to be involved in several processes, including cell proliferation, apoptosis, differentiation and tumour growth. The molecular mechanism of biological activity of KYNA in cancer cells is not fully elucidated and further studies should be undertaken. Due to limited data, it cannot be excluded that KYNA also affects other elements of signalling pathways or cell cycle regulators, and therefore, under certain conditions, supports cancer cell proliferation, motility or survival. KYNA is not only an endogenous constituent of the human body but it is also a component of our daily food. A high content of KYNA was reported in conventional nourishment, such as, honey, broccoli, cauliflower, and potato [25]. The finding that KYNA is easily absorbed from the digestive system and reaches high concentrations in serum and organs [25] seems to be truly significant. Considering the hypothesis of pro-carcinogenous properties of KYNA, immediate significant changes in our eating habits should be recommended. In addition, numerous food products should be strictly avoided.

## KYNA and cancer: directions for future research

Here, we focused on the potential role of KYNA in the process of carcinogenesis and cancer progression. However, accumulated data concerning a relationship between KYNA and cancer are inconsistent and do not provide enough evidence to reach a final conclusion on the role of KYNA in cancer. To clarify the ambiguous role of KYNA in carcinogenesis and cancer progression, large-scale multicentre studies carried out on different levels, in vitro, in vivo and clinical studies, are necessary. Particularly, the following issues should be elucidated:

### Distribution of KYNA in cancer and surrounding tissues

The research on a large group of cancer patients would be of great scientific value. As it was discussed previously, due to various factors affecting the KYNA level in physiological fluids, measurements of this compound in cancer and surrounding tissues seem to be the most relevant. Importantly, in contrast to previous one, the study concerning changes in KYNA concentration between cancer patients and control group should include additional analyses of patients’ diet (low and high KYNA content) and markers of inflammation, which, as it was discussed previously, may influence the KYNA concentration in body fluids.

### Expression of genes coding enzymes of kynurenine pathway and KAT distribution in cancer and surrounding tissues

Searching for correlation between KYNA content, type of cancer and the stage of cancer progression should include not only the changes in the physiological level, but also in the level of genetic alternations. The possible alternations in expression level of genes coding kynurenine pathway enzymes have not been excessively studied in cancer so far.

### Cancer progression in animals with KAT overexpression and KAT knockout animals

Genetic modifications, including knockout animals, animals with KAT or KMO overexpression, may introduce significant changes in KYNA level in particular organs or the entire organism and may result in a better understanding of the role of KYNA in the cancer promotion and progression. Interestingly, due to fast life cycle, simplicity of genetic modifications and well-established cancer models, zebrafish may be one of the most valuable animal models [116, 117]. Additionally, the animal model gives the opportunity to deeply investigate the relationship between KYNA, carcinogenesis and the activity of the immune system.

### Dynamics and scheme of KYNA changes during cancer promotion and progression and potential changes during cancer therapy

If KYNA promotes carcinogenesis and cancer progression, its supplementation and production should be decreased or inhibited. Decrease of KYNA supply in human daily diet seems not to be difficult, as only a few food products contain a significant amount of this compound, including chestnut honey and other honey, bee products, fresh broccoli and potato [25, 118]. On the other hand, decrease of endogenous KYNA production may be also achieved by administration of KATs inhibitors. Although potent inhibitors of KATs are currently tested in the treatment of some brain disorders including Alzheimer’s disease and dementia, the use of KAT inhibitors in anticancer therapy has not been studied [119, 120]. Both strategies are possible for immediate implementation.

Assuming anticancer activity of KYNA, there are many theoretical possibilities to introduce KYNA as a supportive agent to anticancer therapy. Taking into consideration the knowledge about KYNA content in food products [25, 118], KYNA supplementation may be easily increased by diet modification. Interestingly, the anticancer potential of Mediterranean diet, considered rich in KYNA, has been already postulated [121]. Moreover, KYNA level in cancer tissue may be easily increased locally by direct application and may be used topically in the skin, oral cavity, eye, vagina or bladder. It should be noted that KYNA has been previously administrated to the human skin by topical application of cream with no observed side effects [122]. KYNA may be also administered orally leading to increase of KYNA level in gastrointestinal tract. In addition, it is easily absorbed and distributed to peripheral organs [25]. KYNA can also be administered intravenously even at high doses due to its low toxicity. Previous studies revealed that KYNA was non-toxic for rodents after continuous 6-h-long intravenous infusion of tested compound in a dose of 100 mg/kg/h [123]. Similarly, KYNA administered in this mode could be used during the cycle of chemotherapy. Importantly, there are some studies that confirmed the beneficial effect of KYNA on anticancer activity of drugs commonly used in standard chemotherapy [37, 71]. However, further studies are necessary to verify whether cancer tissue may accumulate KYNA or not and therefore, to choose the most effective mode of administration in anticancer therapy. Potential use of KYNA in therapy of brain tumours, especially glioblastoma, needs special attention. In vitro experiments did not clarify whether KYNA has a pro-carcinogenic or antiproliferative potential [37, 62]. Importantly, KYNA does not cross the brain–blood barrier and continuous long-lasting subdural infusion of high doses of KYNA increased the risk of myelin damage and myelin loss [124].

There is also a possibility that KYNA does not exert any activity during cancer promotion and progression and we should consider whether the previous results show specific effects of KYNA activity or non-specific effects of modified cancer cells’ metabolism. KYNA is only one of the elements of kynurenine pathway. Kynurenine pathway produces several biologically active metabolites and plays various functions in the organism including detoxification of excess tryptophan and control of its plasma availability, regulation of hepatic heme biosynthesis, regulatory role in organism via niacin and NAD^+^ synthesis [3]. Kynurenine pathway directly affects metabolic activity and in the same way may affect metabolism of cancer cells. All important redox cofactors NAD^+^ and NADP^+^ play the crucial role in various essential cellular processes, including energy metabolism, cell proliferation, DNA repair and apoptosis [3, 125]. Depletion of NAD^+^ leads to inhibition of glycolysis, activity of citric acid cycle and oxidative phosphorylation, thus, the process of NAD^+^ synthesis is an attractive molecular target for anticancer therapy. Previous studies revealed that depletion of NAD^+^ in cancer cells results in dysfunction of antioxidant defense system, inhibition of proliferation and apoptosis interacting with MAPK and p53 signalling pathways [125]. It cannot be excluded that differences in KYNA concentrations in physiological fluids in cancer patients vs. healthy control group may not prove any KYNA activity towards the tumour cells, but the observed effect is only the body’s response to the ongoing cancer process. Importantly, experimentally increased KYNA concentration may also affect the balance of biochemical reactions and in a non-specific manner KYNA may change the activity of cancer cells. The genetic manipulations or chemical inhibition of downstream reactions of kynurenine pathway leading to changes in NAD^+^ level without any impact on KYNA synthesis would clarify whether KYNA or disruption of NAD^+^ synthesis plays a crucial role in cancer promotion and progression. Taking those into consideration, the potential use of KYNA as a diagnostic/prognostic marker in carcinogenesis or cancer therapy should be excessively investigated. Unfortunately, there are no studies describing the dynamics of changes in the level of KYNA and other KYN metabolites during the cancer treatment so far.

Summing up, there is a need for further studies and new data which will help to solve the dilemma of the role of KYNA in cancer in an unambiguous manner.

## Abbreviations

3-HAA: 3-Hydroxyanthranilic acid
AFMID: Kynurenine formamidase (arylformamidase)
AhR: Aryl hydrocarbon receptor
alpha7 nAChR: α-7 nicotinic acetylcholine receptor
BCRP: Breast cancer resistance protein
BMSCs: Bone marrow stromal cells
BRAF: B-Raf proto-oncogene, serine/threonine kinase
ERK: Extracellular signal-regulated kinases
GPR35: G protein-coupled receptor 35
HAAO: 3-Hydroxyanthranilate 3,4-dioxygenase
hOAT: Human organic anion transporter
IDO: Indoleamine 2,3-dioxygenase
KAT: Kynurenine aminotransferase
KMO: Kynurenine 3-monooxygenase
KRAS: KRAS proto-oncogene
KYN: Kynurenine
KYNA: Kynurenic acid
KYNU: Kynureninase
MAPK: Mitogen-activated protein kinase
MDS: Myelodysplastic syndrome
MGUS: Monoclonal gammopathy of undetermined significance
MM: Multiple myeloma
MRP: Multidrug resistance protein
NMDA: *N*-Methyl-d-aspartate
NSCLC: Non-small cell lung carcinoma
PI3K/Akt: Phosphoinositide 3-kinase/protein kinase B
PIK3CA: Phosphatidylinositol-4,5-bisphosphate 3-kinase catalytic subunit alpha
PTEN: Phosphatase and tensin homolog
QPRT: Quinolinate phosphoribosyl transferase
QUIN: Quinolinic acid
RCC: Renal cell carcinoma
SCC: Squamous cell carcinoma
TDO: Tryptophan 2,3-dioxygenase
TP53: Tumour protein p53
